# Diagnostic challenges in acute monoblastic/monocytic leukemia in children

**DOI:** 10.3389/fped.2022.911093

**Published:** 2022-09-28

**Authors:** Elena Varotto, Eleonora Munaretto, Francesca Stefanachi, Fiammetta Della Torre, Barbara Buldini

**Affiliations:** Pediatric Hematology Oncology and Stem Cell Transplant Division, Maternal and Child Health Department, Padua University, Padua, Italy

**Keywords:** pediatric acute monoblastic/monocytic leukemia, immunophenotype, FAB-M5 AML, children, multiparametric flow cytometry, cytogenetics, molecular biology, cytomorphology

## Abstract

Acute monoblastic/monocytic leukemia (AMoL), previously defined as M5 according to FAB classification, is one of the most common subtypes of Acute Myeloid Leukemia (AML) in children, representing ~15–24% of all pediatric AMLs. Currently, the characterization of monocytic-lineage neoplasia at diagnosis includes cytomorphology, cytochemistry, immunophenotyping by multiparametric flow cytometry, cytogenetics, and molecular biology. Moreover, measurable residual disease (MRD) detection is critical in recognizing residual blasts refractory to chemotherapy. Nonetheless, diagnosis and MRD detection may still be challenging in pediatric AMoL since the morphological and immunophenotypic features of leukemic cells potentially overlap with those of normal mature monocytic compartment, as well as differential diagnosis can be troublesome, particularly with Juvenile Myelomonocytic Leukemia and reactive monocytosis in infants and young children. A failure or delay in diagnosis and inaccuracy in MRD assessment may worsen the AMoL prognosis. Therefore, improving diagnosis and monitoring techniques is mandatory to stratify and tailor therapies to the risk profile. This Mini Review aims to provide an updated revision of the scientific evidence on pediatric AMoL diagnostic tools.

## Introduction

Acute monoblastic/monocytic leukemia (AMoL) belongs to the heterogeneous group of Acute Myeloid Leukemias (AMLs), accounting for about 15–24% of AMLs in children (1–3).

The French-American-British (FAB) classification defines AMoL as AML-M5 in the presence of at least 30% of blast cells in bone marrow (BM) or peripheral blood (PB) specimens, 80% or more of those belonging to the monocytic lineage, generally evaluated on Wright-Giemsa or May-Grünwald-Giemsa-stained smears (4–6). The 2016 Revision of the World Health Organization (WHO) classification of myeloid neoplasms and acute leukemias lowers the level of blast cells defining an AML to 20%, at least 80% of those being of monocytic derivation, and includes AMoL in the category *AML, not otherwise specified (NOS)* (1). Regardless, AMoL morphology has been described as associated with specific genetic abnormalities not included in the WHO classification yet, as well as genetic alterations belonging to the category *AML with recurrent genetic abnormalities*, which prevail on morphological and immunophenotypic features for the final AML group assignment, as per WHO classification (1).

According to morphological features and maturation stage of monocytic-lineage blast cells, AMoL may be further divided into acute monoblastic leukemia (AML-M5a as per FAB classification) when at least 80% of the monocytic cells are monoblasts and acute monocytic leukemia (AML-M5b as per FAB classification) when blasts at a more mature stage are prevalent (predominantly promonocytes) (4–6).

From a clinical point of view, AMoL is characterized by high leukocyte counts (7–10), major propensity for extramedullary infiltrates as compared with other AMLs, involving skin, gum, and central nervous system (11–18), and possible association with disseminated intravascular coagulation along with other bleeding disorders (19–21).

Since leukemic cells morphology and immunophenotypic features may substantially overlap with those of normal monocytic compartment, AMoL diagnosis and Measurable Residual Disease (MRD) detection are challenging.

Nowadays, the main diagnostic tools for monocytic cell compartment characterization include standard cytomorphology and cytochemistry, immunophenotyping by multiparametric flow cytometry (MFC), cytogenetics, and molecular biology (22, 23).

This review highlights standard diagnostic tools in AMoL.

### Standard cytomorphology

The morphological evaluation is the first step of the process that leads to AMoL diagnosis.

Both PB and BM smears are traditionally stained with May-Grünwald-Giemsa or Wright-Giemsa stains, allowing proper cytosolic granules and nuclear chromatin discrimination. Cytomorphology allows detecting monocytic lineage cells at different maturation stages (monoblasts, promonocytes, immature or abnormal monocytes, mature monocytes, and reactive monocytes) as well as blast cells. Nevertheless, in clinical practice, the discrimination of blast cells from normal precursors or reactive monocytes is troublesome due to their relevant morphological overlap.

In the presence of a morphological picture suggesting AMoL, monoblasts and promonocytes should be considered as blasts/blast equivalents in acute monoblastic (M5a) and monocytic (M5b) leukemia.

In acute monoblastic leukemia (M5a), BM is usually hypercellular, showing a predominant population of large (up to 30 μm in diameter), poorly differentiated blasts with a rounded to oval nucleus containing reticular and immature chromatin pattern and one to four light-blue nucleoli. The cytoplasm is usually abundant and basophilic, with rare scattered azurophilic granules, fine vacuolizations, and the absence of Auer rods. The presence of translucent pseudopod formation may erroneously be misinterpreted as a double membrane (24–26).

In acute monocytic leukemia (M5b), promonocytes predominate in BM specimens, whereas mature monocytes are prevalent in PB samples. Extramedullary lesions can be composed of both two cell types. Promonocytes have less basophilic cytoplasm with a grayish ground-glass appearance and occasional large azurophilic granules and vacuoles (27). They have large, irregular-shaped, and folded nuclei, often containing nucleoli, with nuclear segmentations. This aspect allows differentiating them from monoblasts (Table 1) (28). Auer rods are rare. Associated hemophagocytes (erythrophagocytosis) may be observed, sometimes in case of positivity for *t* (8; 16) (p11.2; p13.3)/KAT6A-CREBBP translocation (1, 28, 29). Mature monocytes are usually characterized by a large overall size, deeply folded or convoluted nuclei with condensed, mature-appearing chromatin, absence of prominent nucleoli, and abundant gray-blue cytoplasm, often with a few vacuoles and azurophilic granules (Table 1) (28).

**Table 1:** Recommendations for monocyte evaluation in the blood or bone marrow smears by Goasguen et al. (28).

	**Nuclear shape**	**Chromatin**	**Cytoplasm**	**Comments**
Monoblast	Round/oval	Delicate/lace-like Nucleolus prominent	Basophilic Rare azurophilic Granules	Large: 20–30 μm
Promonocyte	Convoluted/indented	Delicate/lace-like Nucleolus prominent	Variably basophilic Variable azurophilic Granules	Except for nuclear shape, very similar to monoblast
Immature monocyte	Convoluted/indented	More condensed Rare nucleolus	Less basophilic than promonocyte or blast, but more basophilic than mature monocyte	Resemble monocytes but less mature and smaller
Monocyte	Lobulated/indented	Condensed No visible nucleolus	Gray Occasional azurophilic granules Occasional vacuoles	Large: 20–25 μm

Immature monocytes, also defined as “abnormal” or “atypical” monocytes, can be found in normal BM smears as well as in Chronic Myelomonocytic Leukemia (CMML) and Juvenile Myelomonocytic Leukemia (JMML) (30). They are similar but smaller and more basophilic than mature monocytes, showing immature-appearing chromatin, prominent nuclear folds or convolutions, and, rarely, small nucleoli (Table 1) (28).

Reactive monocytes, usually encountered in response to inflammatory or infectious disorders [systemic lupus erythematosus, rheumatoid arthritis, sarcoidosis, Epstein-Barr virus (EBV), cytomegalovirus (CMV), human herpes virus-6 (HHV-6), histoplasma, mycobacteria, and toxoplasma] can show a range of morphologic patterns, including variable cell size (12–20 μm), increased nuclear to cytoplasmic ratio, less condensed or immature chromatin, small nucleoli, and prominent cytoplasmic vacuolization, basophilia, and granularity (Table 1) (28).

Therefore, morphological AMoL diagnosis requires expert operators, constant inter- and intra-laboratory training, updating, and confrontation, to minimize interobserver variability and standardize the final interpretation of blood smears.

### Cytochemistry

Cytochemistry represents a helpful tool in discriminating immature monocytic- from myeloid-lineage cells and identifying monocytic cells at different maturation stages.

The myeloperoxidase (MPO) staining, a fundamental tool of the diagnostic process, is usually negative in immature monocytic cells (monoblasts) and mature monocytes, whereas promonocytes may show slight and scattered positivity. Conversely, granulocytic cells typically show strong MPO positivity (1, 29).

Intense non-specific esterase activity (NSE) [naphthyl acetate esterase, naphthol AS-D acetate esterase (CAE), and alpha-naphthyl butyrate esterase] represents one of the most specific hallmarks of cytochemical staining in monocytic lineage leukemia, resulting strongly positive in both pathological monoblasts and promonocytes, even if 10–20% of AMoL cases show NSE negativity or weak positivity. Moreover, NSE is negative or only weakly positive in myeloid-lineage cells and allowing their discrimination from monocytic ones (1, 29).

Nevertheless, NSE requires a complex and time-consuming multistep preparation and is associated with variability in staining results and subsequent interpretation. Therefore, it should be routinely performed only in laboratories with expert morphologists to obtain accurate results.

The addition of sodium fluoride to NSE staining allows further discriminating monocytic- from myeloid-lineage cells since it inhibits NSE reaction only in the first group (1, 31).

Regarding other cytochemical stainings, used only exceptionally in the clinical routine process, Sudan black and naphthol AS-D chloroacetate esterase are typically negative in monoblasts, whereas tartrate-sensitive acid phosphatase is usually positive. Variable results are described with the periodic acid-Schiff stain, 3-glucuronidase, and oil red O stain (24–26, 32–35).

### Immunophenotyping by multiparametric flow cytometry

**Figure 1:**
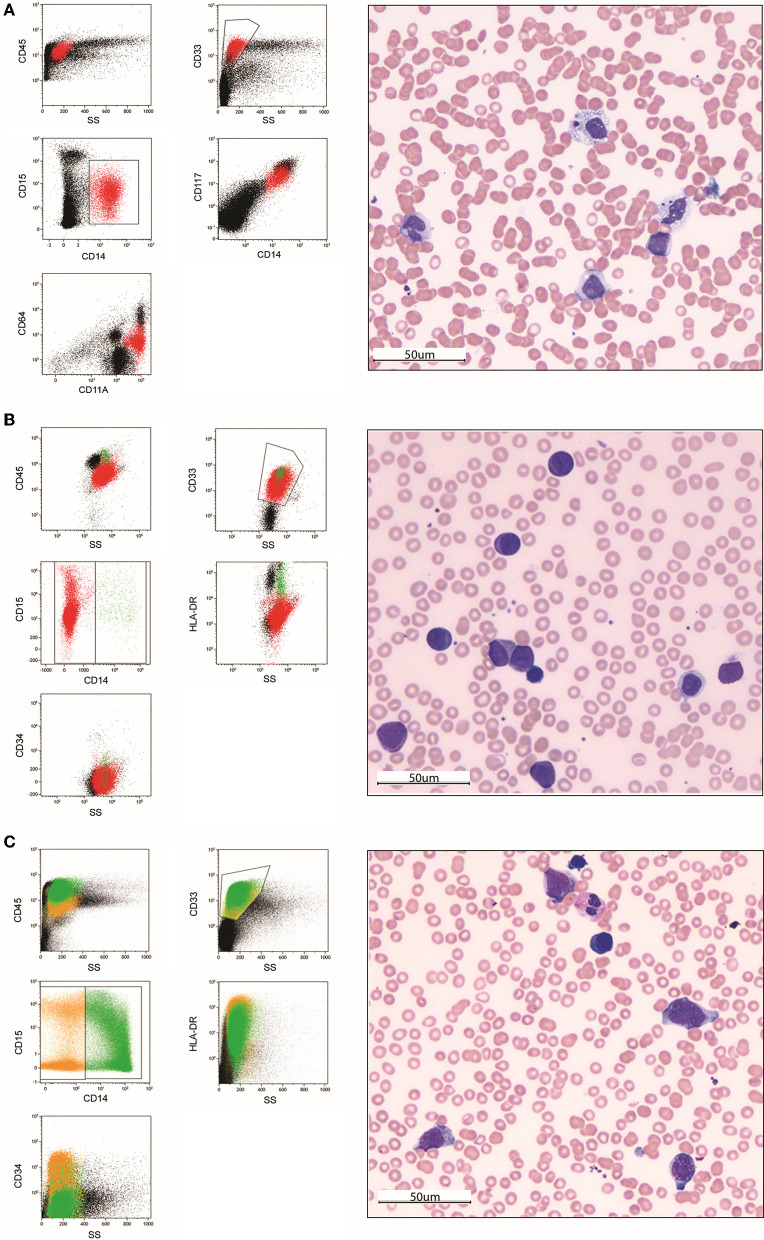
Morphological and immunophenotypic analysis of two pediatric acute monoblastic/monocytic leukemia cases at diagnosis **(A,B)** and after the first induction course **(C)**. In the first case **(A)**, immunophenotyping at diagnosis on a bone marrow sample allows identifying a population of blast cells characterized by an asynchronous expression of early myelomonocytic-lineage markers CD33, CD117, and CD64 together with the maturing monocytic-lineage marker CD15 and the mature monocytes marker CD14. The May-Grünwald-Giemsa (MGG)-stained bone marrow smear (40 × magnification) shows a population of large cells with abundant grayish ground-glass cytoplasm containing rare vacuoles and irregular-shaped and folded nuclei with nucleoli. Two images of hematophagocytosis are also present. In the second case at diagnosis **(B)**, blast cells show an immunophenotype overlapping with immature monocytic-lineage cells (positivity of CD33 and CD15, negativity of CD14) in the absence of CD34 expression. The MGG-stained smear shows a homogeneous population of large, poorly differentiated blasts with a rounded nucleus, reticular chromatin pattern and one to multiple nucleoli, and basophilic cytoplasm, including several azurophilic granules (monoblasts). After the first induction course **(C)**, standard morphology identifies a population of immature monocytes not clearly discriminable from blast cells. Conversely, MFC-MRD analysis is able to define them as regenerating cells and exclude the presence of residual blasts. (40 × magnification). Color legend: red (monoblasts), green (monocytes), orange (monocyte precursors).

Immunophenotyping by MFC accurately detects and characterizes any potential pathological cells in biological samples. In the presence of an abnormal proliferation of monocytic-lineage cells, at diagnosis MFC is critical in discriminating reactive from dysplastic or leukemic origin. Indeed, it allows overcoming the well-known overlap existing between monocytic-lineage blasts cells and normal counterparts, identifying asynchronous monocytic marker combinations as well as any aberrant expression of myeloid and lymphoid markers (Figure 1). Immunophenotyping at diagnosis relies on a careful selection of monoclonal antibodies (MoAb) conjugated with specific fluorochromes to be used in multiple combinations (36–38).

Several markers are useful to discriminate monocytic precursors at different maturation stages and monocytic-lineage blasts from myeloid counterpart, among those the most significant are: CD45, CD11a, CD38, CD99 (pan-leukocytic markers) (38–41); CD34 (hematopoietic stem cells marker) (42, 43); CD64, CD13, CD33, CD123, cyMPO, CD117 (early myelomonocytic-lineage markers) (43, 44); CD11b, CD15, CD36 (maturing monocytic-lineage markers); CD14, CD4, CD35, CD300e (mature monocytes markers); HLA-DR (pan-monocytic lineage marker) (43, 45); cyLYZO (pan-myelomonocytic lineage marker) (46); CD66b (47), CD16 (maturing and mature granulocytic-lineage marker) (48); CD371 (granulocyte-macrophage-lineage marker) (49). Additionally, a complete immunophenotypic study requires association with B- (CD19, CD10, cyCD79A, cyCD22) and T-(CD7, CD3, CD2, CD56, CD4, CD8, CD45RA) lymphoid- lineage markers (38, 43, 50, 51).

Regarding normal precursors, monoblasts are characterized by strong positivity of CD34, CD117, and HLA-DR and progressive upregulation of CD64 (Fc receptor/Fc gamma receptor 1). Even if CD117 is a tyrosine kinase receptor widely expressed on hematopoietic, endothelial, and immature cardiomyocytes, its association with CD34 and CD64 accurately identifies monoblasts precursors. Maturing promonocytes keep strong positivity of CD64 and HLA-DR but show a progressive downregulation of CD34 and CD117 and acquisition of CD14 (belonging to the family of leucine-rich repeat proteins), CD36 (fatty acid translocase/platelet glycoprotein 4/glycoprotein IIIb/glycoprotein PAS-4/scavenger receptor class B member 3), and CD35 (C3b-C4b receptor/complement receptor type 1). Finally, mature monocytes present strong positivity of CD64, CD14, and CD35, downregulation of HLA-DR, negativity of CD34 and CD117, and acquisition of CD300c (CMRF35-like molecule 6). Unlike CD64 and CD14, CD36, CD35, and CD300c are not mainly committed to the monocytic lineage. Regardless, they play a crucial role in identifying monocytic precursors in the described associations (43).

At diagnosis, the knowledge of monocytic maturation sequence of antigen expression critically helps detect AMoL blast cells, potentially escaping from this schema. In acute monoblastic leukemia, blasts may show a more immature immunophenotype, presenting a heterogeneous positivity of CD34, strong positivity of CD64 and HLA-DR, downregulation of maturating monocytic-associated markers CD36 and CD11b, and absence of mature monocytic-associated antigens CD14, CD35, and CD300c, together with downregulation of myelomonocytic markers cyMPO, CD13, CD123. Conversely, acute monocytic leukemia may be associated with a more mature immunophenotype, characterized by the expression of mature monocytic-associated markers CD14, CD35, and CD300c and the absence of CD34 (43).

AMoL blast cells may show aberrant expression of markers like CD56, CD7, CD19, cyCD79a (lymphoid antigens), and NG2 (neural/glial antigen 2), the positivity of which is potentially associated with the presence of KMT2A rearrangements. Whether the expression of NG2, CD7, CD19, and cyCD79a on monocytic lineage cells undoubtfully indicates the presence of blast cells (43, 52–54), CD56 may be aberrantly positive on reactive and normal monocytes (55–57).

An accurate definition of blast immunophenotype at diagnosis is also critical for measurable residual disease monitoring by MFC (MFC-MRD) during therapy. MFC-MRD relies on the Leukemia-Associated Immunophenotype (LAIP) approach, in which leukemic blasts immunophenotype are characterized at diagnosis and tracked at re-evolution points, and the Different-from-Normal (DfN) approach, based on discrimination between cells with aberrant immunophenotypes and normal counterpart during follow-up (58).

In AMoL, MFC-MRD is usually more challenging than in other AML subtypes. First, the blast population may have an immunophenotypic heterogeneity at diagnosis potentially hidden by a predominant LAIP. Second, blast immunophenotype may change during therapy, hampering the accuracy of the LAIP method. Third, in the absence of aberrant lymphoid marker expression, AMoL blast LAIPs frequently overlap the immunophenotype of monocytic-lineage precursors in regenerating BM, making MFC-MRD detection very challenging. Therefore, the DfN approach may be more useful in these cases, even if more complex and requires a deep knowledge of normal hematopoietic precursors immunophenotypic maturation curves (58–66).

Consequently, it is critical to identify new markers specific for monocytic-lineage blasts and possibly not expressed on normal hematopoietic cells. In the last few years, several antigens have been studied to detect AMoL blasts. In 2015, Pereira and colleagues demonstrated the aberrant expression of B lymphoid-lineage antigen CD37, a transmembrane protein of the tetraspanin superfamily, in 15 different AML cell lines, 5 of those AMoL, and confirmed it in a cohort of 26 patients'-derived AML samples (67). In 2019, Lo et al. showed the positivity of antigen CD302, a type I transmembrane C-type lectin receptor usually expressed on myeloid-lineage cells, in all the 6 AMoL out of 33 analyzed AML samples (68). CD157, a glycosylphosphatidylinositol-anchored glycoprotein, is another marker associated with AMoL, but also with normal myelomonocytic lineage cells (69). Additionally, in 2020 Churchill et al. described the coexpression of the two inhibitory leukocyte Ig-like receptors, LILRB1 and LILRB4, belonging to a family of immunoregulatory receptors, specific for AMoL (70).

To summarize, MFC is an essential step of AMoL diagnosis in childhood, allowing blasts discrimination from normal monocytic counterpart even when their cytomorphological definition is challenging. In diagnostics practice, an accurate MFC diagnosis of AMoL relies on the following steps: (i) negativity of B- (at least 2 among CD19, CD10, cyCD22, and cyCD79) or T-lineage (s/cyCD3 and CD7) acute lymphoblastic leukemia criteria; (ii) blasts assignment to the myeloid/monocytic lineage (positivity of at least 2 among MPO, CD13, CD33, CD64, CD65, CD117, cy-LYZO, CD14, CD11c); (iii) investigation of specific monocytic-lineage markers (CD11b, CD15, HLA-DR, CD371), (iv) hematopoietic stem cell marker (CD34), and (v) potential aberrant expression of CD7, CD56, CD19, cyCD79a, NG2 (1, 38, 71).

### Cytogenetics and molecular studies

In the last few decades, genetic characterization has shown to be critical in AML clinicopathological evaluation, refining diagnosis, assigning patients to a risk class, predicting prognosis, and promoting target therapy (72, 73). Genetic characterization mainly relies on cytogenetic (conventional karyotyping and FISH analysis) and molecular studies [next-generation sequencing (NGS) and real-time reverse transcriptase-polymerase chain reaction (RT-PCR)] (72).

Pediatric AMoL is a heterogeneous disease as concerns genetics and is associated with different outcomes regarding the displayed aberrations. At cytogenetic analyses, it presents a normal karyotype in about 20% (74). Among somatic cytogenetic aberrations, chromosomal translocations involving the KMT2A gene on 11q23 are the most frequent, accounting for about 20% of the genetic anomalies in AMoLs (74). Previously known as MLL (Mixed Lineage Leukemia), the KMT2A gene encodes an important histone-H3 lysine-4 (H3K4) methyltransferase involved in the epigenetic regulation of hematopoietic stem/progenitor cells development.

A total of 135 KMT2A rearrangements have been described in leukemias, mostly resulting in KMT2A-fusion proteins capable of transforming hematopoietic stem cells into leukemic blasts with stem cell-like properties (72, 75–78). The most frequent fusion partners in AMLs are AF9/MLLT3, AF10/MLLT10, ELL, ENL/MLLT1, AF6/MLLT4, and MLL-PTDs (77, 79). Nowadays, only AML with *t* (9; 11) (p21.3; q23.3)/KMT2A-MLLT3 is included in the category *AML with recurrent genetic abnormalities* of the WHO classification (1).

Of note, Balgobind et al. reported a significantly better prognosis of *t* (9; 11) (p22; q23) positive AMLs in the presence of M5 FAB rather than non-M5 FAB. Moreover, Balgobind et al. described new risk subgroups related to KMT2A translocations: *t* (1; 11) (q21; q23) showed favorable outcomes regardless of other risk factors, whereas *t* (6; 11) (q27; q23), *t* (10; 11) (p12; q23), and *t* (10; 11) (p11.2; q23) turned out to be an independent risk factor of poor clinical outcome (80).

Another clinically relevant genetic aberration in AMoLs, not detectable with cytogenetic studies, is the FMS-like tyrosine kinase 3 (FLT3) gene mutation, described in about 5.5–26.5% of AMoLs in different pediatric cohorts (71, 74, 81–83). FLT3 belongs to the class III tyrosine kinase receptor family expressed on hematopoietic progenitors and promotes cell survival, proliferation, and differentiation being activated by an extracellular ligand (FLT3 ligand) (71).

FLT3 mutations may be found in normal karyotype AMLs and associated with additional cytogenetic lesions. They are classified into two major groups: internal tandem duplications (ITDs-about two-thirds of FLT3 mutations) and point mutations in the tyrosine kinase domain (TKD-about one-third of FLT3 mutations), both leading to the constitutive activation of the FLT3 kinase. The prognostic role of FLT3-TKD remains unclear, whereas FLT3-ITDs have been associated with an unfavorable prognosis, particularly in the presence of a high allelic ratio (> 0.51) (1, 74, 84–91). Regardless, it was recently demonstrated that the association with additional genetic aberrations, like WT1 and NPM1 mutations and NUP98 translocations, modulates the outcome (91).

AMoL may be also rarely associated with Core Binding Factor (CBF) mutations involving chromosome 16 [inv (16) (p13.1q22) and *t* (16; 16) (p13.1; q22)/CBFB-MYH11], and *t* (8; 21) (q22; q22.1); RUNX1-RUNX1T1, both included in the category *AML with recurrent genetic abnormalities* according to WHO classification (1, 3, 91, 92). To note, the presence of any CBF abnormalities allows diagnosing an AML independently from blast count (1).

AMoL could also be associated with somatic mutations of the nucleophosmin gene 1 (NPM1), encoding a nuclear pleiotropic protein that regulates cell growth and proliferation, protein chaperoning, maintenance of genomic stability, and activation of tumor suppressor p53. NPM1 mutations involve about 6.5% of pediatric AMLs, may be associated with normal karyotype and FLT3/ITD mutations, and show a wide range of morphological subtypes, of those FAB-M4 and M5 are the most frequent (93–95). NPM1 mutations seem to confer a favorable outcome in childhood AML, in the absence of FLT3-ITD mutations (91, 95).

Finally, AMoL may display activating mutations of NRAS and KRAS genes, encoding two G-proteins involved in proliferation and survival signals transmission among cells (96). NRAS and KRAS mutations are detectable in about 18–20% and 28% of pediatric AMoLs, respectively, being more common in younger patients (74, 96). RAS mutations may be associated with normal karyotype, as well as with other genetic aberrations, like NPM1 mutation and MLL-PTD (91, 96). The prognostic role of RAS mutation is still unclear, being variable in different pediatric and adult cohorts (96).

### Immunophenotypic and molecular targets for precision medicine

Beyond their critical role in pediatric AMoL diagnostics, MFC and molecular biology allow identifying potential targets for a precision medicine approach. Among MFC-detectable markers, CD33 is the target of the antibody-drug conjugated gemtuzumab ozogamicin, approved by the FDA in 2018 for the treatment of relapsed/refractory pediatric AMLs (97) and adopted in several first- and additional-line pediatric therapeutic trials (98–104). CD123-positive AMoLs may potentially benefit from flotetuzumab, a CD123/CD3 bispecific antibody, and antibody-drug conjugated IMGN632 and tagraxofusp-erzs, all under investigation for pediatric relapsed/refractory AMLs (105, 106). Additionally, CD33, together with CD123 and CD371, is under investigation for generating chimeric antigen-receptor T cells (CAR-T) directed against AML (106–108).

Among molecular alterations, AMoL harboring FLT3-ITD mutation may benefit from targeted therapy with FLT3 inhibitors (sorafenib, midostaurin, sunitinib, lestaurtinib, quizartinib, gilterinib) (109, 110). In the presence of KMT2A rearrangements, AMoL may potentially benefit from targeted therapy with DOT1L-inhibitor (pinometostat) and menin-inhibitors (KO-539, SNDX-5613), actually under investigation (109, 111, 112).

## Conclusions

An accurate characterization of monocytic-lineage cells in PB and BM samples is crucial to avoid misdiagnosis. Indeed, AMoL needs to be differentiated from other morphological AMLs subtypes (AML without maturation, AML with minimal differentiation, acute megakaryoblastic leukemia, acute myelomonocytic leukemia, microgranular acute promyelocytic leukemia, CMML, JMML), and reactive causes of monocytosis.

Moreover, several infections can result in myelomonocytosis along with persistent fever, failure to thrive, hepatosplenomegaly, skin lesions, anemia, and thrombocytopenia, mimicking leukemia.

Developing new diagnostic markers is required to obtain unequivocal discrimination between leukemic and reactive monocytes. Particularly, the identification of new specific MFC antigens may help recognize monocytic blasts even in the absence of specific leukemia-related genetic markers.

## Author contributions

BB and EV designed the study. EM, FS, FD, EV, and BB wrote the manuscript. All authors contributed to the article and approved the submitted version.

## Funding

This project is part of a training activity supported by the residency program in Pediatrics of the Department of Woman and Child's Health, University of Padua, Padua, Italy.

## Conflict of interest

The authors declare that the research was conducted in the absence of any commercial or financial relationships that could be construed as a potential conflict of interest.

## Publisher's note

All claims expressed in this article are solely those of the authors and do not necessarily represent those of their affiliated organizations, or those of the publisher, the editors and the reviewers. Any product that may be evaluated in this article, or claim that may be made by its manufacturer, is not guaranteed or endorsed by the publisher.
